# Cross-sectional study of lipoprotein(a) and the severity of coronary artery disease, cerebrovascular disease, and peripheral vascular disease in a group of South Asian patients

**DOI:** 10.1097/XCE.0000000000000327

**Published:** 2025-03-27

**Authors:** Pandula M. Athauda-arachchi, Lalindra Kaththiriarachchi, Wickrama Salgado, Sulakkana De Silva, Tiyara Salgado, Mumtaz Farooq, M Javid Jibran, Yumandi Godakanda Arachchi, Samidi Premanath, Sithira L. Senaratne, Sanjalee P. Samarakoon, Hiran Rathnayake, Mariam Shajahan

**Affiliations:** a General Sir John Defence University; b Durdans Hospital Colombo, Colombo, Sri Lanka

**Keywords:** atherosclerosis, cerebrovascular disease, coronary disease, Lp(a) level, peripheral vascular disease, SYNTAX score

## Abstract

**Background:**

Atherosclerotic cardiovascular diseases (ASCVD), including coronary artery disease (CAD), cerebrovascular disease (CVD), and peripheral vascular disease (PVD), remain the leading cause of death globally. South Asians exhibit a higher incidence of cardiovascular diseases than other ethnicities, attributed to a range of genetic, environmental, and lifestyle factors. Lipoprotein(a) [Lp(a)] with a unique apolipoprotein(a) component, has emerged as a marker of atherosclerosis and ASCVD risk, with evidence to promote arterial plaque formation and thrombogenesis.

**Objective:**

The aim of this study was to explore the associations between Lp(a) levels and the severity of CAD, CVD, and PVD in a group of South Asian patients.

**Methods:**

Following ethical approval, 60 consecutive patients who underwent coronary angiography for any indication were reviewed. There were 51 eligible participants who were evaluated for Lp(a) level, Synergy Between Percutaneous Coronary Intervention with Taxus and Cardiac Surgery (SYNTAX) score, and severity of CVD and PVD. The SYNTAX-I score was calculated using two-observer consensus on coronary angiograms. Assessment of CVD was by ultrasound/Doppler, and PVD by estimating ankle-brachial index using Doppler. The multisite arterial disease score 2 (MADS2) and SYNTAX score tertiles were used to group the patients. Statistical analysis was performed using the SPSS software.

**Results and discussion:**

In this group, we identified a statistically significant difference with higher Lp(a) levels being associated with more severe coronary disease (SYNTAX tertile 2,3). Despite a numerical trend, statistical significance was not confirmed for Lp(a) levels in relation to MADS2-CVD or MADS2-PVD scores. A larger study may be required to assess these aspects.

## Introduction

Atherosclerosis is a multifactorial inflammatory disease that affects medium and large arteries and is primarily driven by lipid accumulation [1]. Atherosclerotic cardiovascular disease (ASCVD) is the predominant cause of death worldwide [2]. ASCVD includes coronary artery disease (CAD), cerebrovascular disease (CVD) [3], and peripheral vascular disease (PVD) [4]. According to data published by the Global Burden of Diseases and risk factors study, the prevalence of CAD, CVD, and PVD in 2019 was 197, 101, and 113 million, respectively [5].

Southeast Asia represents 23% of the global population and has a substantially higher risk of cardiovascular disease than other ethnicities [2]. Research indicates a higher prevalence of CAD and early onset myocardial infarction in South Asians than other populations [6]. Worldwide estimates indicate that the greatest number of patients with PAD are in Southeast Asia, and the Western Pacific regions are asymptomatic [7]. In South Asian countries, demographic changes, urbanization, and increased exposure to major stroke risk factors increase the stroke burden [8]. Furthermore, evidence demonstrates that the South Asian region has a high prevalence of stroke, especially in younger individuals [9].

According to the health profile of Sri Lanka (www.worldlifeexpectancy.com), based on the WHO, Centers for Disease Control and Prevention, World Bank, and United Nations statistics published in 2020, deaths because of coronary heart disease have reached 22.66% of total deaths [10]. Furthermore, the prevalence of CAD is increasing, leading to high hospital mortality rates in Sri Lanka and difficulties in providing costly advanced cardiac care to prevent end-stage heart failure [11]. Studies conducted in Sri Lanka indicate a concentration of risk factors in metropolitan areas and higher socioeconomic groups, with an increasing prevalence among younger individuals. The burden of stroke in Sri Lanka is steadily increasing [12]. According to the health profile of Sri Lanka (www.worldlifeexpectancy.com) 2020, Stroke deaths accounted for 5.86% of the total deaths. Sri Lankan statistics on PVD are scarce [13]. PVD is predicted to become an upcoming public health concern in Sri Lanka, particularly the aging population [13].

The discernible increase in the incidence of CAD, CVD, and PVD among the South Asian population, particularly at a younger age, highlights the urgency of exploring novel markers for early detection and intervention [14]. The rise is attributed to a spectrum of risk factors: lifestyle choices [15], stress [16], BMI [17], meal patterns [18], environmental elements such as air pollution [19,20], and genetic factors [21], including dyslipidemia [22] and thrombophilia [23].

The burden of risk factors, such as hypertension, diabetes, and dyslipidemia, cannot be overstated. Hypertension, as the most significant modifiable cardiovascular risk factor, is responsible for a substantial portion of all strokes (48%), and a significant proportion of coronary events (18%) [24]. Dyslipidemia, on the other hand, is associated with the highest population-attributable risk for myocardial infarction (49.2%) [25]. The occurrence of silent ischemia is significantly higher in individuals with diabetes, ranging from 10 to 20%, in contrast to those without diabetes, where it is found in only 1–4% of cases [26].

The impact of these risks is modified by favorable lifestyle modifications and new and improved drugs, such as lipid-lowering, antiplatelet, and anticoagulation drug therapies. However, the increasing prevalence of cardiovascular diseases persists with a significant gap in the treatment of ASCVDs [27].

For diagnosing CAD, the previous emphasis was on exercise testing [28] but with ongoing technological advancements, international guidelines have changed with the shift of emphasis [29] to invasive or noninvasive imaging to diagnose CAD and inform the choice of appropriate therapies, emphasizing risk scoring followed by appropriate advanced imaging modalities [30]. On the basis of the pretest probability, it is decided whether to perform computed tomography, MRI with perfusion [31], stress echocardiography, single-photon emission computed tomography [32], and Fractional flow reserve with invasive coronary angiography [33], while invasive coronary angiography remains the gold standard [34].

Ultrasound and MRI play pivotal roles [35–38], with advancing spatial resolutions enhancing diagnostic capabilities [39] for the detection of CVD. However, a growing population presents with cardiovascular diseases at a younger age or without traditional risk factors [40].

Our research focused on lipoprotein (a) [Lp(a)], a novel marker that has garnered attention for its association with cardiovascular diseases [41]. Lp(a), is a low-density lipoprotein (LDL)-like particle that contains apolipoprotein(a) in addition to apolipoprotein B [42]. The genetic basis of Lp(a) levels is complex, and involves both environmental and genetic factors. Elevated levels of Lp(a) are considered a risk factor for ASCVDs [43–45]. Similar to LDL, Lp(a) undergoes oxidation. Oxidized lipoproteins are more likely to be retained in arterial walls, contributing to the development of atherosclerosis. Retained lipoproteins can elicit an inflammatory response and promote the formation of foam cells, a hallmark of atherosclerotic plaques [46]. Foam cells are usually macrophages in which the cytoplasm is filled with cholesterol [47].

As patients with seemingly favorable lipid profiles can manifest cardiovascular diseases, it is imperative to delve into other lipid components, such as Lp(a) and their impact on cardiovascular health [48,49]. This research aimed to comprehensively analyze the role of Lp(a) in association with CAD, CVD, and PVD, considering its potential significance in identifying individuals at risk who may not be captured by traditional risk assessments. The fact that a significant portion of the younger population suffer from ASCVDs despite having normal lipid profiles highlights the limitations of current risk assessment methods.

The potential role of Lp(a) as a target for the treatment of cardiovascular diseases is of significant pharmacological interest. Research in this area could lead to the development of new therapeutic interventions and medications, while drugs, such as PCSK9 inhibitors, are increasing. Genomic variations in Lp(a) add an intriguing layer of complexity that offers opportunities for personalized medicine. The observation that the Sri Lankan coronary arteries are generally small and that obstruction can occur more rapidly in such individuals is an important population-specific factor. This factor underscores the importance of considering unique demographic characteristics in cardiovascular risk assessments. Investigating Lp(a) may help to address this issue. Through this, we aimed to contribute valuable insights into the field, facilitating early detection, targeted interventions, and an enhanced understanding of cardiovascular disease etiology.

### Objective

The aim of this study was to determine the association between Lp(a) levels and the severity of CAD, CVD, and PVD in a selected group of patients.

## Methods

### Study design

This was a cross-sectional study conducted at the Cardiac Catheter Laboratory of Durdans Hospital, Colombo, Sri Lanka, over a period of 6 weeks.

### Ethics approval

This study was approved by the Ethics Review Committees of Durdans Hospital and Kotelawala Defence University (approval no. RP/2024/07).

### Study population

The study included consecutive individuals undergoing coronary angiography for any clinical indication at Durdans Hospital, with participants providing consent for additional blood sampling and vascular assessments.

#### Sample size

A total of 60 patients meeting inclusion criteria were enrolled.

#### Inclusion criteria

Patients of any sex and age presenting for coronary angiography at Durdans Hospital Colombo, consenting to participate in the study.

#### Exclusion criteria

Patients were unwilling to provide informed consent or individuals with contraindications to the diagnostic procedures.

### Data collection and measurements

The following measurements were recorded. Lp(a) measurement: venous blood samples were collected and analyzed for Lp(a) levels using a quantitative method (ELISA). CAD assessment: CAD using the online Synergy Between Percutaneous Coronary Intervention with Taxus and Cardiac Surgery (SYNTAX) score calculator [50], derived from the coronary angiography findings of two experienced cardiologists. CVD was evaluated via carotid duplex ultrasound, whereas PVD was assessed through the ankle-brachial index using Doppler ultrasound by an experienced radiologist.

### Statistical analysis

To address the mildly skewed distribution of Lp(a) data, a log transformation was applied to approximate normality. The Shapiro–Wilk test confirmed the normality of the log-transformed Lp(a) levels, yielding a test statistic of approximately 0.98 with a *P* value of 0.432. This validation permitted the use of parametric tests, including Welch’s independent sample *t* test and analysis of variance (ANOVA) on Lp(a) data, alongside graphical analyses such as boxplots, with comparisons made across various groupings [e.g. SYNTAX tertiles, multisite arterial disease score 2 (MADS2-CVD), MADS2-PVD and composite scores] [51].

## Results

### Descriptive statistics

The number of patients according to age, sex, coronary disease SYNTAX tertile, MADS2-CVD score, MADS2-PVD score, and composite score (SYNTAX tertile + MADS2-CVD and MADS2-PVD) are shown in Table 1.

**Table 1:** Distribution of subjects according to age, sex, coronary disease SYNTAX tertile, MADS2-CVD score, MADS2-PVD score, and composite score (SYNTAX tertile + MADS2-CVD and MADS2-PVD)

Category	Group	Count (*n*)
Sex	Male	36
	Female	15
Age groups	30–49	11
	50–69	25
	70–89	14
	90+	1
SYNTAX tertile-CAD	SYNTAX tertile-CAD 0,1	26
	SYNTAX tertile-CAD 2,3	25
MADS2-CVD	MADS2-CVD 0	17
	MADS2-CVD 1,2	34
MADS2-PVD	MADS2-PVD 0	17
	MADS2-PVD 1,2	34
Composite scores	Composite 0,1,2	27
	Composite 3,4,5	24

The mean, median, and SD of the Lp(a) levels for the age groups are shown in Table 2.

CAD, coronary artery disease; CVD, cerebrovascular disease; Lp(a), lipoprotein(a); MADS2, multisite arterial disease score 2; PVD, peripheral vascular disease; SYNTAX, Synergy Between Percutaneous Coronary Intervention with Taxus and Cardiac Surgery.

**Table 2:** Mean, median, and SD of the lipoprotein(a) levels by age group

Age group (years)	Mean Lp(a) (mg/dl)	Median Lp(a) (mg/dl)
30–49	25.70 ± 7.75	21.8
50–69	31.69 ± 3.9	30.0
70–89	31.25 ± 2.82	28.0
90+	17.00	17.0

**Fig. 1:**
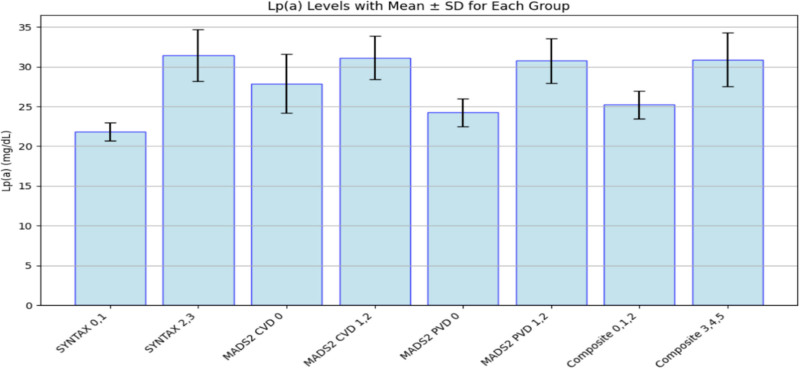
Mean and SD of Lp(a) levels in patients grouped by SYNTAX/MADS2/composite scores. CVD, cerebrovascular disease; Lp(a), lipoprotein(a); MADS2, multisite arterial disease score 2.

The mean, median, and SD of the Lp(a) levels for each group are shown in Table 3, and the visual representation in Fig. 1.

Lp(a), lipoprotein(a).

**Table 3:** Mean, median, and SD of the lipoprotein(a) levels by coronary or vascular disease scores

Group	Mean Lp(a) (mg/dl)	Median Lp(a) (mg/dl)
SYNTAX tertile-CAD 0,1	21.83 ± 1.13	20.0
SYNTAX tertile-CAD 2,3	31.44 ± 3.28	29.0
MADS2-CVD 0	27.88 ± 3.70	26.0
MADS2-CVD 1,2	31.14 ± 2.74	28.5
MADS2-PVD 0	24.24 ± 1.77	21.2
MADS2-PVD (1,2)	30.75 ± 2.78	29.0
Composite score 0,1,2	25.24 ± 1.75	21.8
Composite score 3,4,5	30.89 ± 3.36	28.0

CAD, coronary artery disease; CVD, cerebrovascular disease; Lp(a), lipoprotein(a); MADS2, multisite arterial disease score 2; PVD, peripheral vascular disease; SYNTAX, Synergy Between Percutaneous Coronary Intervention with Taxus and Cardiac Surgery.

### Statistical analysis

Summary of findings: these findings are summarized in Table 4.

**Table 4:** Statistical difference of lipoprotein(a) levels by coronary or vascular disease groups

Analysis	Group comparison	*t* Statistic (Welch)	*P* Value	Significance
SYNTAX tertile analysis	0,1 vs. 2,3	2.90	0.008	Statistically significant
Composite score groups	0,1,2 vs. 3,4,5	1.34	0.201	NS
MADS2-CVD	0 vs. 1,2	−0.69	0.493	NS
MADS2-PVD	0 vs. 1,2	1.60	0.144	NS

CVD, cerebrovascular disease; MADS2, multisite arterial disease score 2; PVD, peripheral vascular disease; SYNTAX, Synergy Between Percutaneous Coronary Intervention with Taxus and Cardiac Surgery.

SYNTAX tertile analysis: a statistically significant difference in Lp(a) levels between the SYNTAX tertiles exists, suggesting that the severity of CAD may be influenced by Lp(a) levels.

MADS2-CVD: the comparison of the MADS2-CVD groups showed no significant difference in Lp(a) levels, suggesting that the presence of CVD does not correlate with Lp(a) in this cohort.

MADS2-PVD: similarly, the MADS2-PVD comparison indicates no significant difference in Lp(a) levels, further suggesting that PVD severity may not be directly related to Lp(a) levels.

Composite score groups: no significant difference was observed in Lp(a) levels between the composite score groups, indicating that these scores may not be predictive of Lp(a) levels in this study.

### Analysis of variance

An ANOVA test across all groups (composite 0,1,2 vs. 3,4,5; MADS2-CVD 0 vs. 1,2 and SYNTAX tertile 0,1 vs. 2,3) yielded an *F* statistic of 0.71 with a *P* value of 0.620, suggesting no significant difference in Lp(a) levels across all groups taken collectively. This may require a large number of patients to demonstrate these differences.

**Fig. 2:**
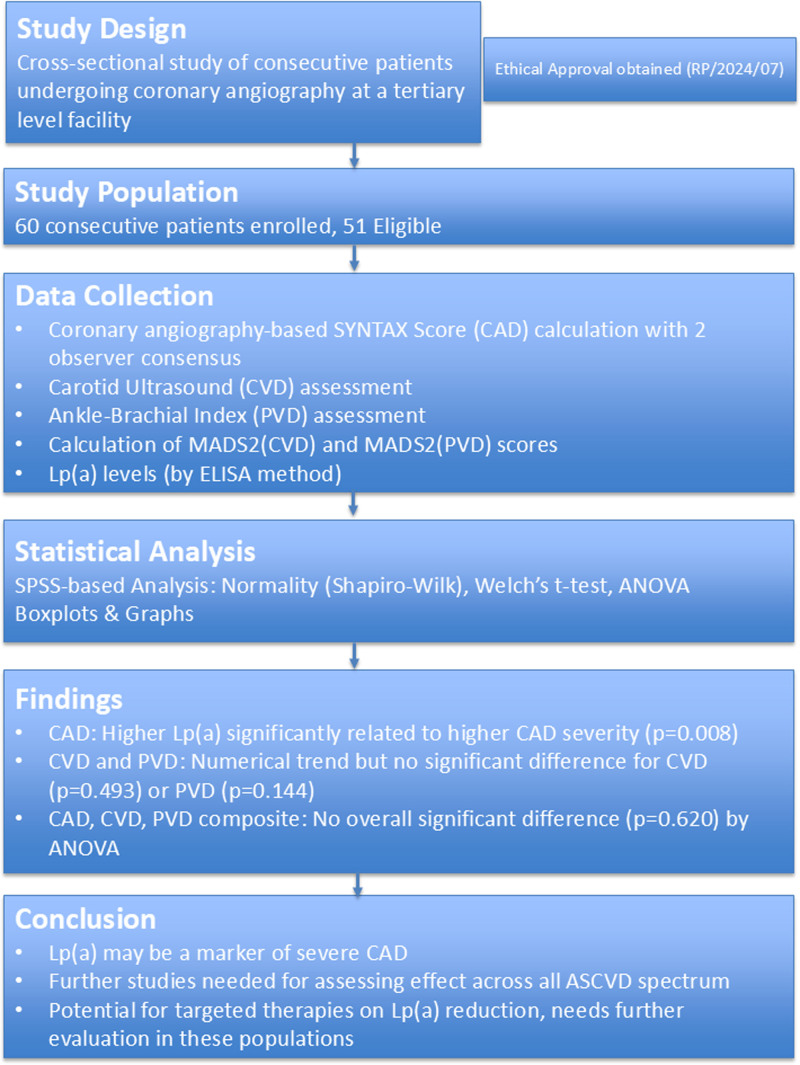
Central illustration: study flowchart. ANOVA, analysis of variance; ASCVD, atherosclerotic cardiovascular diseases; CAD, coronary artery disease; CVD, cerebrovascular disease; Lp(a), lipoprotein(a); MADS2, multisite arterial disease score 2; PVD, peripheral vascular disease; SYNTAX, Synergy Between Percutaneous Coronary Intervention with Taxus and Cardiac Surgery.

The study flow and key results have been summarized in Fig. 2 as the central illustration. A Summary of key learning points are stated in Table 5.

**Table 5:** Major summary points of the study

1.	Lp(a) is considered a potential independent risk factor of accelerated atherosclerosis
2.	Scanty data is available on Lp(a) in South Asian patients, who appear to develop atherosclerotic vascular disease early, sometimes in spite of a ‘normal’ conventional lipid profile.
3.	In this group, a statistically significant difference was seen with Lp(a); higher levels were associated with more severe coronary disease (SYNTAX tertile 2,3)
4.	In this group, a numerical trend was seen but a statistical significance was not confirmed for Lp(a) levels in relation to CVD or PVD (as assessed with MADS2-CVD or MADS2-PVD scores). Larger study is warranted

CVD, cerebrovascular disease; Lp(a), lipoprotein(a); MADS2, multisite arterial disease score 2; PVD, peripheral vascular disease; SYNTAX, Synergy Between Percutaneous Coronary Intervention with Taxus and Cardiac Surgery.

## Discussion

Our analysis identified a statistically significant difference in Lp(a) levels across the SYNTAX tertile groups, with higher Lp(a) levels associated with more severe CAD (SYNTAX tertile 2,3). The SYNTAX tertiles are clinically significant, as they categorize patients into levels of CAD complexity, providing insights into the severity and prognosis of CAD.

In this study, the association between high Lp(a) levels and increased SYNTAX tertile complexity highlighted the potential role of Lp(a) as a marker of disease severity. These findings support the utility of Lp(a) in risk stratification, suggesting that patients with high Lp(a) and SYNTAX scores may require more aggressive management and follow-up. This also suggests that in South Asian patients, pharmacological measures to control Lp(a) levels may play a role in preventing the development of aggressive coronary disease.

However, no significant differences were found in Lp(a) levels in relation to MADS2-CVD, MADS2-PVD, or composite scores. This suggests that while Lp(a) may play a role in CAD severity, it did not correlate strongly with other vascular diseases in this cohort. The weak correlations and lack of significant predictors in the multivariate model underscore the need for further research with many subjects, potentially including other contributing variables, to better understand the role of Lp(a) in ASCVD.

## Conclusions

The findings from this study suggest that Lp(a) levels are significantly associated with CAD severity, but may not correlate with the severity of CVD or PVD. Our findings are in line with recent publications from the European Atherosclerosis Society, suggesting that Lp(a) may indeed represent a causal risk factor for ASCVD, as well as aortic valve stenosis, with heterogeneity observed in different ethnic groups [52]. Further research with larger, longitudinal datasets could provide greater insights into the relationship between Lp(a) and ASCVD in South Asian populations and provide comparisons to European data to help determine whether reducing Lp(a) may reduce the rising ASCVD burden in such populations.

## Acknowledgements

The authors thank Durdans Hospital Colombo, General Sir John Defence University, and all study participants.

No industrial funding was obtained. P.M.A.-A. provided personal funding for tests.

P.M.A.-A was in-charge of the patient care in all cardiology procedures, study design, data analysis, manuscript writing, and submission. L.K. was involved with conceptualization, literature review, and ERC submissions. W.A.S. is the radiologist for providing cerebrovascular disease/peripheral vascular disease assessments. C.D.S. prepared data analysis tables, ERC documentation, patient and clinical coordination, and vetting of scoring. M.J.J., M.F., T.S., and Y.G. were medics recruiting patients for the study and tabulated data. S.M.S.P.S., B.A.S.L.S., N.V.S.S.P., H.M.B.R., and M.S. interviewed and collected patient data, performed the literature search, and drafted initial documentation.

Ethical approval was obtained from the regulatory body after submitting all documents – Ethics review committee of KDU (approval no. RP/2024/07).

Consent for publication was obtained for anonymized data.

Data were available on request.

### Conflicts of interest

There are no conflicts of interest.
